# CD51 distinguishes a subpopulation of bone marrow mesenchymal stem cells with distinct migratory potential: a novel cell-based strategy to treat acute myocardial infarction in mice

**DOI:** 10.1186/s13287-019-1439-y

**Published:** 2019-11-20

**Authors:** Dong-Mei Xie, Yuan-Long Li, Jie Li, Qinglang Li, Guihua Lu, Yuansheng Zhai, Juhong Zhang, Zhibin Huang, Xiuren Gao

**Affiliations:** 1 Department of Cardiology, The First Affiliated Hospital, Sun Yat-sen University, Guangzhou, 510080 China; 2 NHC Key Laboratory of Assisted Circulation (Sun Yat-sen University), Guangzhou, 510080 China; 3 Zhongshan School of Medicine, Sun Yat-sen University, Guangzhou, 510080 China

**Keywords:** CD51, Mesenchymal stem cell, Migration, Myocardial infarction, Inflammation

## Abstract

**Background:**

Experimental and clinical trials have demonstrated the efficiency of bone marrow-derived mesenchymal stromal/stem cells (bMSCs) in the treatment of myocardial infarction. However, after intravenous injection, the ineffective migration of engrafted bMSCs to the hearts remains an obstacle, which has an undesirable impact on the efficiency of cell-based therapy. Therefore, we attempted to identify a marker that could distinguish a subpopulation of bMSCs with a promising migratory capacity.

**Methods:**

Here, CD51-negative and CD51-positive cells were isolated by flow cytometry from Ter119^−^CD45^−^CD31^−^bMSCs and cultured in specifically modified medium. The proliferation ability of the cells was evaluated by 5-ethynyl-2′-deoxyuridine (EdU) staining or continuously monitored during culture, and the differentiation potential was assessed by culturing the cells in the appropriate conditioned media. Wound healing assays, transwell assays and quantitative polymerase chain reaction (qPCR) were used to measure the migratory ability. The mice were subjected to a sham operation or myocardial infarction (MI) by permanently occluding the coronary artery, and green fluorescent protein (GFP)-labelled cells were transplanted into the mice via intravenous infusion immediately after MI. Heart function was measured by echocardiography; infarct myocardium tissues were detected by triphenyl tetrazolium chloride (TTC) staining. Additionally, immunofluorescence staining was used to verify the characteristics of CD51^+^bMSCs and inflammatory responses in vivo. Statistical comparisons were performed using a two-tailed Student’s *t* test.

**Results:**

In this study, the isolated CD51^−^bMSCs and CD51^+^bMSCs, especially the CD51^+^ cells, presented a favourable proliferative capacity and could differentiate into adipocytes, osteocytes and chondrocytes in vitro. After the cells were transplanted into the MI mice by intravenous injection, the therapeutic efficiency of CD51^+^bMSCs in improving left ventricular ejection fraction (LVEF) and left ventricular fractional shortening (LVFS) was better than that of CD51^−^bMSCs. Compared with CD51^−^bMSCs, CD51^+^bMSCs preferentially migrated to and were retained in the infarcted hearts at 48 h and 8 days after intravenous injection. Accordingly, the migratory capacity of CD51^+^bMSCs exceeded that of CD51^−^bMSCs in vitro, and the former cells expressed higher levels of chemokine receptors or ligands. Interestingly, the retained CD51^+^bMSCs retained in the myocardium possessed proliferative potential but only differentiated into endothelial cells, smooth muscle cells, fibroblasts or cardiomyocytes. Transplantation of CD51^+^bMSCs partially attenuated the inflammatory response in the hearts after MI, while the potential for inflammatory suppression was low in CD51^−^bMSC-treated mice.

**Conclusions:**

These findings indicated that the CD51-distinguished subpopulation of bMSCs facilitated proliferation and migration both in vitro and in vivo, which provided a novel cell-based strategy to treat acute MI in mice by intravenous injection.

## Background

Bone marrow-derived mesenchymal stromal/stem cells (bMSCs) possess a self-renewal capacity and can be expanded to a large scale in vitro [1]. Moreover, bMSCs have multi-differentiation potency and can differentiate into adipocytes, osteocytes and chondrocytes in vitro*.* Increasing evidence suggests that a subpopulation of bMSCs exists and may play a critical role in the homing and healing of injured tissue [2, 3].

Cardiovascular disease is the leading cause of death worldwide [4], and myocardial infarction (MI) accounts for 80% of the mortality in patients with ischaemic heart disease [5]. Although advances in medical and surgical treatment of MI have been achieved, the increasing prevalence and high mortality of heart disease demand a continuous search for innovative treatments [5]. bMSCs are activated and migrate to injured targets. Nevertheless, Schmidt-Lucke et al. proved that only a few endogenous circulating MSCs could migrate to the hearts in virus-negative inflammatory cardiomyopathy patients [6]. Moreover, Hoogduijn et al. did not find that endogenous bMSCs were recruited into the bloodstream in heart transplant patients with an aggressive immune response [7]. Hence, systemic administration of exogenous bMSCs is regarded as a promising strategy to repair the damaged heart and restore cardiac function in patients with ischaemic heart disease.

Both experimental and clinical trials have revealed that MSC-based therapy for MI is safe, moderately improves the LVEF and maintains structural integrity [8–10]. However, the extent of recovery is limited after cell implantation, and the optimal source of cells for cardiac repair remains controversial. Indeed, MI intrinsically enhanced bMSC homing to infarcted areas of the heart after intravenous injection, but the quantity of homed cells was too low to meet the therapeutic requirement [11]. At the same time, most of the infused MSCs were not localized to the infarcted myocardium, based on the evidence that the homing capacity of augmented bMSCs was decreased. Although injection of bMSCs into the peri-infarcted areas or left ventricular cavity could improve the therapeutic effects, these procedures were highly complicated and technical, and they probably induced cardiac damage [12]. Considering that a sufficient number of cells are essential for therapeutic benefits, inefficient migration and transient retention of MSCs in the heart inevitably reduced the therapeutic efficacy. Therefore, the identification of a subpopulation of bMSCs that has a sufficient migratory capacity to migrate to the injured hearts after through intravenous injection and presents a robust therapeutic response to MI is urgently needed.

CD51, also called integrin alpha ν, is a heterodimeric integral membrane protein composed of an extracellular domain, a transmembrane region and cytoplasmic domain [13, 14]. According to Pinho et al., double-positive staining for CD51 and PDGFRα serves as a marker of human bone marrow Nestin^+^ MSCs, and these CD51^+^ PDGFRα^+^ MSCs expand into multipotent haematopoietic stem and progenitor cells due to the abundant expression of haematopoietic stem cell maintenance genes [15]. Moreover, CD51 is expressed in MSCs from other organs or tissues, including the testis, kidney, umbilical cord blood and periodontal tissue [16–20]. Here, we hypothesized that CD51-positive bMSCs may be a crucial subpopulation. Interestingly, the locally or systemically injected bMSCs mainly migrated and stayed in the ischaemic myocardium, and few cells were found in the normal zone, which indicated that the ischaemic tissues were chemoattractive to bMSCs. Accordingly, Sui et al. suggested that cancer stem cells with CD51 expression enhanced tumour initiation and metastatic potential [21]. Much earlier, Brooke et al. demonstrated that human bone marrow-derived mesenchymal stem cells expressed CD51 and chemokine receptors such as CCR1, CCR3, CXCR3, CXCR4 and CXCR6 [22]. Therefore, we speculated that receptors or ligands may be expressed on CD51-positive bMSCs and could induce migration to the targeted ischaemic myocardium.

In this study, we aimed to isolate CD51-negative bMSCs and CD51-positive bMSCs from the bone marrow of 14-day-old mice and determine their self-renewal capacity as well as their multi-differentiation potential. Importantly, the migratory capacity of the candidate cells was studied both in vivo and in vitro using flow cytometry, wound healing assays and transwell assays. After the cells were transplanted into mice with MI, the heart function was assessed by echocardiography, and fibrosis was detected by histological staining.

## Methods

### Animals

Male C57BL/6J mice (*n* = 60) at 12 months of age were purchased from the Animal Center of Sun Yat-sen University and were bred in isolator cages under specific pathogen-free conditions with an ambient temperature of 24 °C, 55–65% relative humidity and a 12-h/12-h light/dark cycle. All animal procedures were reviewed and approved by the Sun Yat-Sen University Institutional Animal Care and Use Committee.

### Isolation and culture of bone marrow mesenchymal cells from mice

The bone marrow of 14-day-old C57BL/6 mice was harvested and passed through a 40-μm cell strainer, yielding single cells. Subsequently, the single-cell suspension was incubated with antibodies specific for CD51-PE (1:200 dilution, BD, USA), CD45-FITC (1:200 dilution, eBioscience, USA), Ter119-PECY7 (1:200 dilution; eBioscience) and CD31-APC (dilution 1:200; eBioscience) at 4 °C for 30 min. CD51^−^CD45^−^Ter119^−^CD31^−^ cells (CD51^−^bMSCs) and CD51^+^CD45^−^Ter119^−^CD31^−^ cells (CD51^+^bMSCs) were sorted using flow cytometry (Influx, BD). Then, the isolated cells were allowed to adhere to culture plates (Corning, USA) in medium described as follows: DMEM/F12 (Gibco, USA) containing 10 ng/ml EGF (Pepro tech, USA), 10 ng/ml bFGF (Pepro tech), 2% B27 (Invitrogen, USA), 0.1 mM β-mercaptoethanol (Invitrogen), 1% l-glutamine (Sigma Aldrich, USA), 1% foetal bovine serum (Gibco) and 100 IU/ml penicillin/streptomycin (Invitrogen). Cells were cultured at 37 °C in a 5% CO_2_ atmosphere and propagated every 2 or 3 days.

### Proliferation assays

Cells at passage 5 were digested and resuspended in the medium described above and seeded in a 96-well plate at a density of 1 × 10^4^ cells per well (passage 6). The plates were incubated in BioTek-lionheart FX (BioTek, USA), and the cultured live cells were continuously monitored for 72 h at 37 °C in a 5% CO_2_ atmosphere. Images were captured every 60 min using a × 4 objective, and the cellular numbers were directly counted by Gen5 software (four replicates per sample). In addition, 5-ethynyl-2′-deoxyuridine (EdU, Thermo Fisher, USA) staining was used to confirm cellular proliferation. Briefly, cells were seeded in 48-well plates at a density of 2 × 10^4^ cells per well (passage 6) in a medium containing 10 μM EdU and incubated for 24 h. Thereafter, the cells were fixed, permeabilized and stained with reaction cocktail for 1 h at 37 °C according to the manufacturer’s protocol. Proliferating cells were identified by red staining in the nucleus, and total cells were stained with DAPI (4′, 6-diamidino-2-phenylindole, blue, Thermo Fisher).

### Cellular differentiation in vitro

To demonstrate the multipotency of CD51^−^bMSCs and CD51^+^bMSCs, we cultured cells with conditioned media that induced differentiation into adipogenic, osteogenic or chondrogenic lineages. Cells were cultured in adipogenic medium (Cyagen, China) for 14 days to induce lipid droplets in the cytoplasm and tested by oil red O staining. Osteogenesis was induced for 10 days in osteogenic medium (Cyagen) and confirmed by Alizarin Red staining. Additionally, the cells were incubated with chondrogenic medium (Cyagen) in centrifuge tubes for 21 days, and differentiation into chondrocytes was verified by toluidine blue staining.

### Reverse transcription and quantitative PCR

Total RNA was extracted from the ventricles of the hearts at different times following the instructions from the manufacturer of the RNAprep Pure Tissue Kit (Tiangen, China). One microgram of total RNA was reverse transcribed to complementary DNA (cDNA) with a RevertAid First Strand cDNA Synthesis Kit (Thermo Fisher). The cDNA templates were used for quantitative PCR (qPCR), and the amplified PCR products were monitored by the fluorescence increase caused by SYBR Green dye (Roche, Indianapolis, USA) binding. The primers for the mouse genes are listed in Table 1. The relative mRNA expression levels were normalized to the endogenous control gene Gapdh.

**Table 1:** Primers used for the PCR analysis

Genes	Forward sequence	Reverse sequence
GAPDH	5′-ACCACAGTCCATGCCATCAC-3′	5′-TCCACCACCCTGTTGCTGTA-3′
PPAR-γ	5′-GTACTGTCGGTTTCAGAAGTGCC-3′	5′-ATCTCCGCCAACAGCTTCTCCT-3′
Adipsin	5′-ACCTGACAGCCTTGAGGACGAC-3′	5′-GGGTTCCACTTCTTTGTCCTCG-3′
Leptin	5′-GCAGTGCCTATCCAGAAAGTCC-3′	5′-GGAATGAAGTCCAAGCCAGTGAC-3′
Osteopontin	5′-GCTTGGCTTATGGACTGAGGTC-3′	5′-CCTTAGACTCACCGCTCTTCATG-3′
Osteocalcin	5′-GCAATAAGGTAGTGAACAGACTCC-3′	5′-CCATAGATGCGTTTGTAGGCGG-3′
PTHR	5′-TGAAGGACGCTGTGCTCTACTC-3′	5′-AGTAGAGGAAGAAGGTCACGGC-3′
Collagen II	5′-GCTGGTGAAGAAGGCAAACGAG-3′	5′-CCATCTTGACCTGGGAATCCAC-3′
Collagen X	5′-GTACCAAACGCCCACAGGCATA-3′	5′-GGACCAGGAATGCCTTGTTCTC-3′
Aggrecan	5′-CAGGCTATGAGCAGTGTGATGC-3′	5′-GCTGCTGTCTTTGTCACCCACA-3′
CCR1	5′-GCCAAAAGACTGCTGTAAGAGCC-3′	5′-GCTTTGAAGCCTCCTATGCTGC − 3′
CCR 2	5′-CAAGTAGAGGCAGGATCAGGCT-3′	5′-GCTGTGTTTGCCTCTCTACCAG-3′
CCR3	5′-CCACTGTACTCCCTGGTGTTCA-3′	5′-GGACAGTGAAGAGAAAGAGCAGG-3′
CCR4	5′-GGACTAGGTCTGTGCAAGATCG-3′	5′-TGCCTTCAAGGAGAATACCGCG-3′
CCR5	5′-CCAAGAGTCTCTGTTGCCTGCA-3′	5′-GTCTACTTTCTCTTCTGGACTCC-3′
CCR6	5′-CTGGTGTAGGCGAGGACTTTCT-3′	5′-ACAGAGCCATCCGAGTCGTGAT-3′
CCR7	5′-AGAGGCTCAAGACCATGACGGA-3′	5′-TCCAGGACTTGGCTTCGCTGTA-3′
CCR8	5′-CTGCGATGTGTAAGGTGGTCTC-3′	5′-CCTCACCTTGATGGCATAGACAG-3′
CCR9	5′-GCCATGTTCATCTCCAACTGCAC-3′	5′-CCTTCGGAATCTCTCGCCAACA-3′
CCR10	5′-TCACAGTCTGCGTGAGGCTTTC-3′	5′-CAGTCTTCGTGTGGCTGTTGTC-3′
CXCR1	5′-CCATTCCGTTCTGGTACAGTCTG-3′	5′-GTAGCAGACCAGCATAGTGAGC-3′
CXCR2	5′-CTCTATTCTGCCAGATGCTGTCC-3′	5′-ACAAGGCTCAGCAGAGTCACCA-3′
CXCR3	5′-TACGATCAGCGCCTCAATGCCA-3′	5′-AGCAGGAAACCAGCCACTAGCT-3′
CXCR4	5′-GACTGGCATAGTCGGCAATGGA-3′	5′-CAAAGAGGAGGTCAGCCACTGA-3′
CXCR5	5′-ATCGTCCATGCTGTTCACGCCT-3′	5′-CAACCTTGGCAAAGAGGAGTTCC-3′
CXCR 6	5′-GCAGGAACACAGCCACTACAAG-3′	5′-GGTTCTTCCTGCCATTGCTCAC-3′
IL6	5′-TACCACTTCACAAGTCGGAGGC-3′	5′-CTGCAAGTGCATCATCGTTGTTC-3′

### Acute MI model and cell transplantation

The mouse model of acute MI was generated by permanently ligating the left anterior descending coronary artery (LAD), approximately 0.3 cm from the origination site, and the ischaemic area was pale. Thereafter, 1 × 10^6^ cells suspended in 500 μl of saline or the same volume of saline (vehicle-treated control) were injected into the blood vessels via the tail vein 30 min after MI.

### Echocardiography

Conventional echocardiography of the left ventricle (LV) of each mouse was performed with a mouse echocardiography system (Vevo 2100 Imaging System, Visual Sonics, Toronto, Canada) equipped with a 30-MHz phased transducer at 21 days after treatment. The following parameters were measured: LVEF, LVFS, LV end-systolic volume (LVESV) and LV end-diastolic volume (LVEDV).

### TTC staining

To differentiate the viable and infarct cardiac tissue, we performed a triphenyl tetrazolium chloride (TTC) assay. TTC solution (Servicebio, China) was prewarmed at 37 °C in the incubator for 30 min. The heart was perfused with 30 ml saline, collected and then placed in a freezer (− 80 °C, refrigerator) until the heart became stiff. Quickly, five sections of 1 mm size were cut from the apex to the bottom and immersed in prewarmed TTC solution at 37 °C for 30 min with shaking every 5 min. Images were immediately acquired by a mobile phone (Xiaomi, China).

### Transfection of the lentiviral GFP vector for tracking

An insert-free vector with a green fluorescent protein (GFP) reporter was used as a cell tracing tool, and LentiLox 3.7 (donated by professor Li) was the lentiviral vector used in this study. Cells were cultured in 6-well plates at a density of 5 × 10^4^ cells/well overnight. After replacing the medium, the concentrated lentiviral particles carrying the GFP reporter gene were incubated with cells at a multiplicity of infection (MOI) of 10 for 24 h. Fluorescence microscopy was used to observe the cells expressing GFP at 72 h of infection.

### Flow cytometry assay

Analyses of MSC marker, GFP-positive cells and inflammatory responses were conducted by flow cytometry. For cultured cells, single cells from the cultured plate were digested with trypsin (0.25%). Cell suspensions were incubated with antibodies against CD105-PE (1:200 dilution, eBioscience) at 4 °C for 30 min. The single-cell suspensions of heart tissue were obtained as follows: The hearts were harvested, cut into pieces and incubated with 5 ml of an HBSS digestion solution containing type II collagenase (300 U/ml; Gabo) and DNase I (100 U/ml; Sigma Aldrich, USA). Then, the tissues along with digestion solution were transferred into purple-cap C tube (Miltenyl Biotec, USA) and homogenized by gentleMACS dissociator (Miltenyl Biotec) for 20 s. The homogenate was incubated at 37 °C for 30 min with shaking and homogenized again as described above. Subsequently, the single-cell suspensions were passed through a 40-μm cell strainer, and pure single cells were yielded. Then, cells were fixed in 4% formaldehyde for 10 min and permeabilized by 0.2% Triton X-100/PBS (HyClone, USA) for 15 min. Finally, the cellular suspensions were selectively labelled with antibodies specific for IL1β-PE (dilution 1:200, eBioscience) to test the inflammatory response. For the GFP-positive cells, they were assessed by FITC-positive staining.

### Cellular scratch assay

Wound healing was assessed using a scratch assay in vitro. Diluted extracellular matrices (1:8, Corning) were preincubated in 96-well plates for 30 min in cell culture incubators. After removing the liquid matrices, 100 μl of cell suspension (1 × 10^5^ cells/ml) was added to the plate and cultured in a complete medium as described above. When the cells grew to a density of 100%, a line was scratched into the cell monolayer on the bottom of the plate with the Scratch Assay Starter Kit (Biotek, USA), creating a cell-free area. After gently removing the debris with phosphate-buffered saline (PBS), the proportion of FBS in the culture medium was reduced to 0.5%. The cells were cultured for 12 h, and images were obtained at 0, 6 and 12 h after scratching. The healing area was calculated as the percentage of the total cell area within the initial wound area. Ten fields from each scratch area were used for analyses.

### Cellular migration assay

The migration ability of GFP-positive cells was determined using a 24-well Millicell hanging cell culture insert (8-μm pore size membrane filters, Merck, USA). First, the lower chamber of the 24-well plate was filled with 600 μl of complete culture medium. Then, 100 μl of a cell suspension (5 × 10^5^ cells/ml) in serum-free medium (containing 0.1% bovine serum albumin) was plated into the rehydrated upper chamber. After 8 h of incubation in the cell culture incubator, the inserts were fixed in 4% paraformaldehyde (PFA) for 20 min and stained with crystal violet for 30 min. The cells remaining on the upper surface of the filters were removed with a cotton-tipped swab. Images were acquired by fluorescence and bright-field microscopy (Biotek, USA). The migrated cells were quantified in five randomly selected fields from three independent experiments at × 100 magnification.

### Histological analyses

The hearts were embedded in OCT (Sakura Finetek, USA) and cut into 10-μm long-axis sections or short-axis sections. Masson trichrome staining was performed according to standard methods and described previously [23]. For the immunofluorescence test, tissue sections or cells were sequentially fixed with 4% formaldehyde for 30 min, permeabilized with 0.2% Triton X-100 (HyClone, USA) for 15 min and blocked in 5% bovine serum albumin for 1 h. Then, the samples were incubated with primary antibody overnight at 4 °C followed by the application of the corresponding secondary antibody application for 1 h at room temperature. The primary antibodies used in this experiment were specific for Ki67 (1:200, Abcam, USA), CD31 (1:200, Abcam), α-actinin (1:500, Sigma), α-SMA (1:200, Abcam) and IL-1β (1:200, Abcam). The secondary antibodies were mouse 594 and Rb594. DAPI was used for nuclear counterstaining. All images were obtained by confocal microscopy (LSM800, Zeiss, Germany) or a Lionheart FX (Biotek). At least three fields were used for quantification.

### Statistical analysis

All results were obtained from at least three independent experiments and are presented as the mean ± SD. Most statistical comparisons were performed using a two-tailed Student’s *t* test (between two groups). *P < 0.05, **P < 0.01 and ***P < 0.001 were considered statistically significant. Analyses were performed, and graphs were generated using GraphPad Prism software 6.01 (San Diego, CA).

## Results

### Isolation and culture of CD51^−^bMSCs and CD51^+^bMSCs

**Fig. 1:**
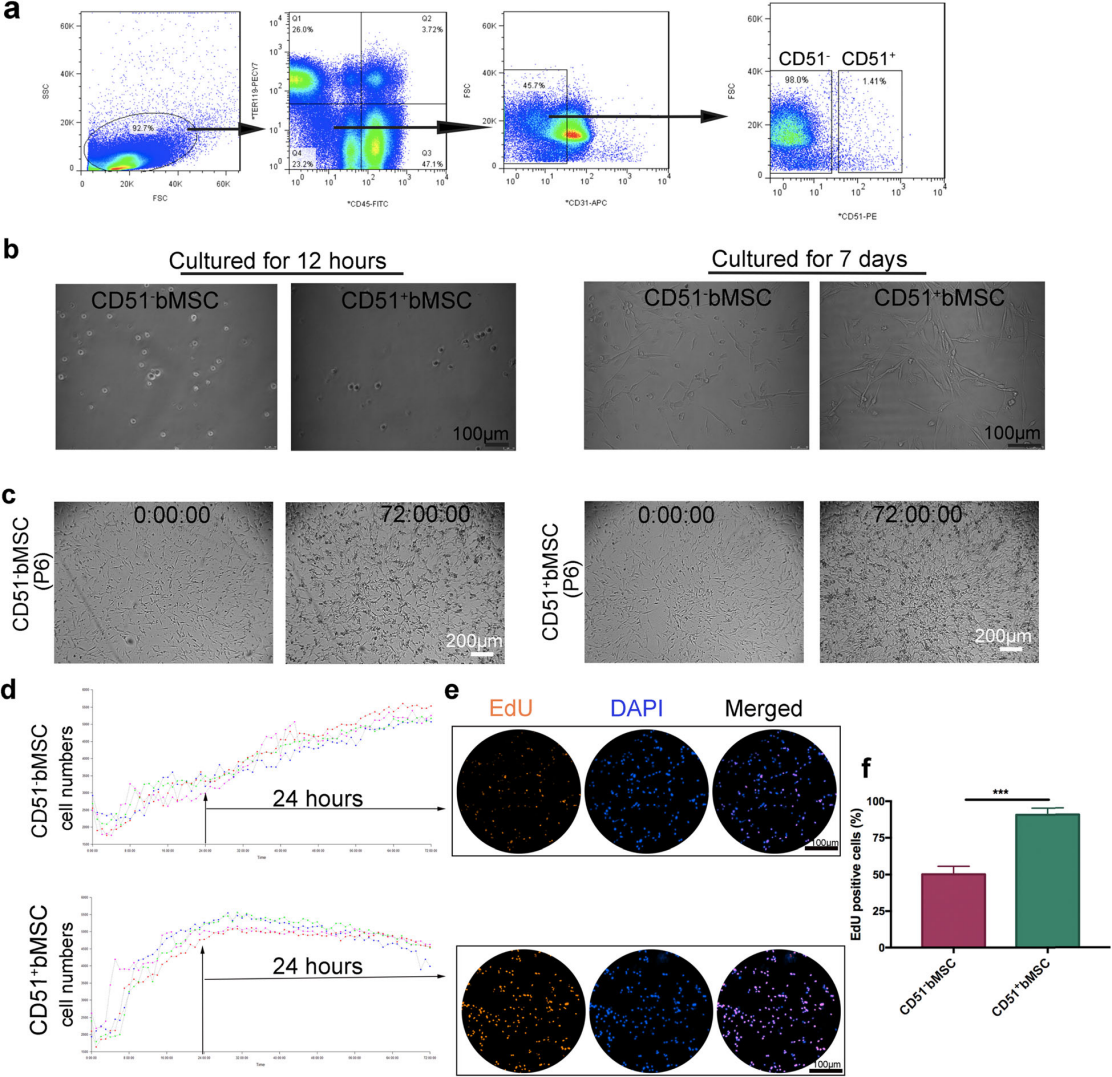
Isolation and culture of CD51^−^bMSCs and CD51^+^bMSCs. **a** CD51^−^bMSCs and CD51^+^bMSCs were isolated using flow cytometry, and the contaminated cells were excluded by the markers CD45, Ter119 and CD31. **b** The isolated cells were cultured in the medium, and images were obtained at 12 h and 7 days. **c** Images of the cells at passage 6 were obtained at 0 h and 72 h after adherence. **d** Proliferation curves based on the cell numbers from 0 to 72 h. **e** Cells were stained with 5-ethynyl-2′-deoxyuridine (EdU) at 24 h. **f** Comparison of EdU-positive cell ratios (*n* = 3). ***P < 0.001. Scale bars are marked in the figure

To obtain the bMSC subgroups from the bone marrow, we used a PE anti-mouse CD51 antibody to isolate the cells through FACS. Simultaneously, antibodies specific for CD45, Ter119 and CD31 were coincubated with CD51 to exclude cell contamination from the haematopoietic, erythroid and endothelial cell lineages, respectively. As shown in Fig. 1a, the CD51^−^bMSCs accounted for approximately 98% of the total CD45^−^Ter119^−^CD31^−^ cells, and the proportion of CD51^+^bMSCs was approximately 1.41%. The freshly isolated cells attached to the dish and became small and round after being cultured for 12 h in the complete medium described above (Fig. 1b). Then, the cells began to flatten out and exhibited a spindle-shaped morphology after being cultured for 7 days. The cells also proliferated to form clusters during this period (Fig. 1b). The cells were subcultured to passage 5 and accumulated for further experiments. At passage 6, we continuously monitored the cells for 72 h in the incubator to compare their proliferative ability (Fig. 1c). The data showed that CD51^+^bMSCs rapidly expanded and almost reached their peak within 24 h, while CD51^−^bMSCs grew slowly, gradually reaching their peak at 70 h (Fig. 1d). To visualize the cell proliferative state, we adopted the EdU assay (Fig. 1e). Similarly, the percentage of EdU-positive cells in CD51^+^bMSCs was significantly higher than that in CD51^−^bMSCs (90.7% ± 2.6% vs 50.2% ± 3.2%, *P* < 0.001) (Fig. 1f). In summary, both CD51^−^bMSCs and CD51^+^bMSCs grew well in the medium after isolation, and they exhibited cellular proliferative activity in vitro, especially CD51^+^bMSCs. In addition, the proliferative advantages of CD51^+^bMSCs were not significantly reduced under hypoxic-ischaemic culture conditions when compared with normoxic conditions when tested by Ki67 staining (Additional file 1: Figure S1a-b). However, TUNEL staining showed that compared with normoxia, hypoxia enhanced the apoptosis of CD51^+^bMSCs (Additional file 1: Figure S1c-d).

### Multi-differentiation capacity of CD51^−^bMSCs and CD51^+^bMSCs

**Fig. 2:**
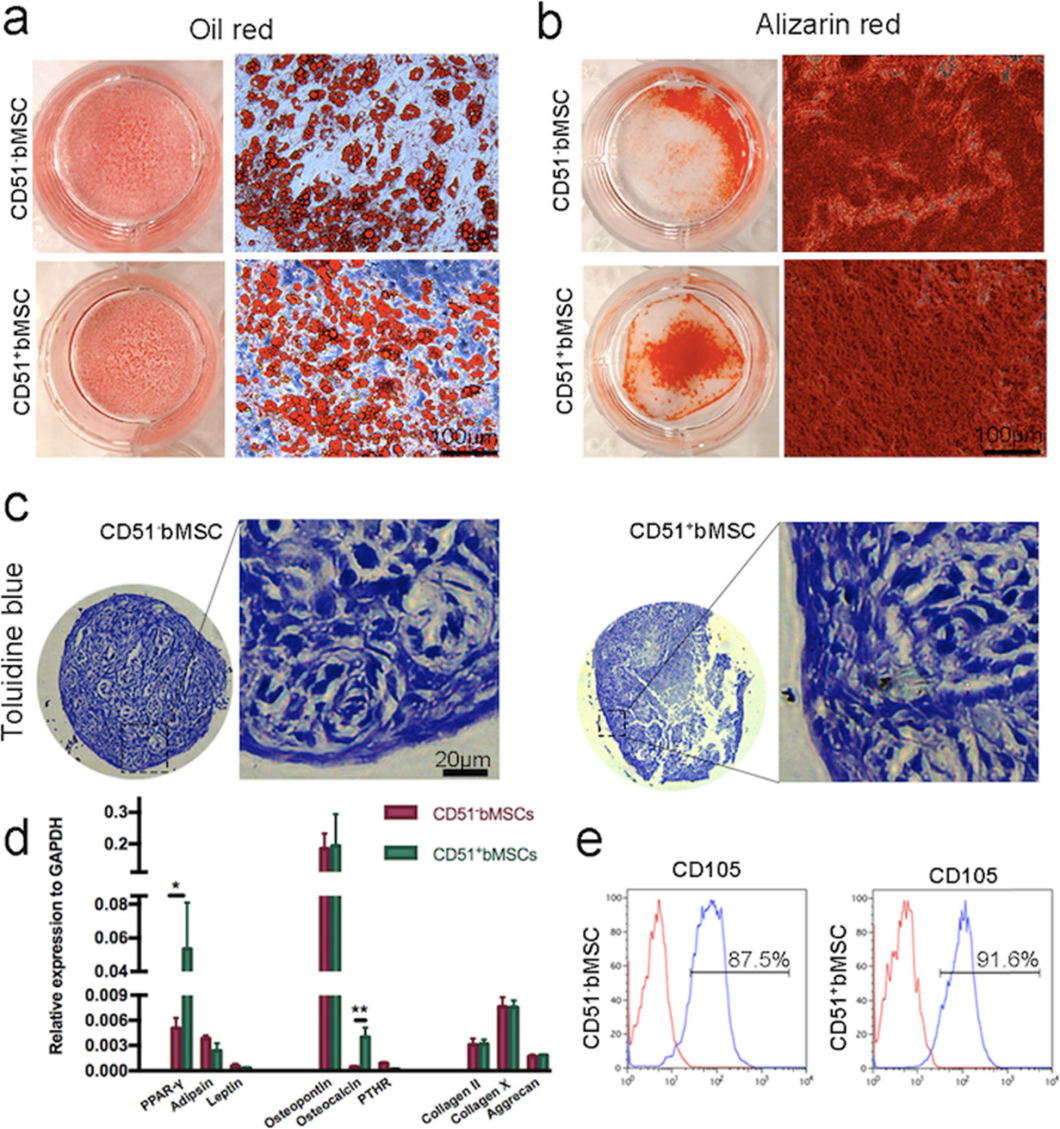
Differentiation of CD51^−^bMSCs and CD51^+^bMSCs. **a** Adipogenic differentiation; cells were stained with oil red O after 14 days of culture. **b** Osteogenic differentiation; cells were stained with Alizarin Red after 14 days of culture. **c** Chondrogenic differentiation; sections of cell balls were stained with toluidine blue after 21 days of culture. **d** Relative mRNA expression of the indicated clusters (adipogenic, osteogenic and chondrogenic); the gene expression levels were normalized to GAPDH (*n* = 3). **e** Representative image of CD105 expression of CD51^−^bMSC and CD51^+^bMSC was determined by flow cytometry. Red histogram is the negative control. The left side of the blue histogram (in the negative control part) represents the negative expression of CD105 marker; the right side represents the positive expression of CD105 marker. Data in all panels are presented as the mean ± SD, *P < 0.05, **P < 0.01. Scale bars are marked in the figure

Given the proliferative capacity of the two groups of cells, we also investigated the multilineage differentiation ability in conditioned media that is favourable for adipogenesis, osteogenesis and chondrogenesis. Large numbers of lipid droplets were detected using oil red O staining both CD51^−^bMSCs and CD51^+^bMSCs, indicating a strong adipogenic differentiation capacity (Fig. 2a). The two groups of cells were also differentiated into abundant osteocytes and were calcified when stained using Alizarin Red (Fig. 2b). According to the toluidine blue staining data, CD51^−^bMSCs and CD51^+^bMSCs also markedly differentiated into chondrocytes and formed cartilaginous nodules (Fig. 2c). In addition, the gene expression of adipocytes, osteocytes and chondrocytes was verified, including the expression of PPAR-γ, adipsin, leptin, osteopontin, osteocalcin, PTHR, collagen II, collagen X and aggrecan. No significant difference was observed in the expression of the indicated genes except for PPAR-γ and osteocalcin (Fig. 2d). CD105 was an accept marker of MSCs, and we studied the expression of CD105 by flow cytometry (Fig. 2e). Our results have shown that positive expression of CD105 in CD51^−^bMSCs and CD51^+^bMSCs were about 87% and 91%, respectively, which indicated almost all cells expressed CD105. Thus, both CD51^−^bMSCs and CD51^+^bMSCs exhibited strong multi-lineage differentiation ability in adipogenesis, osteogenesis and chondrogenesis.

### Cell transplantation improved cardiac function after AMI

**Fig. 3:**
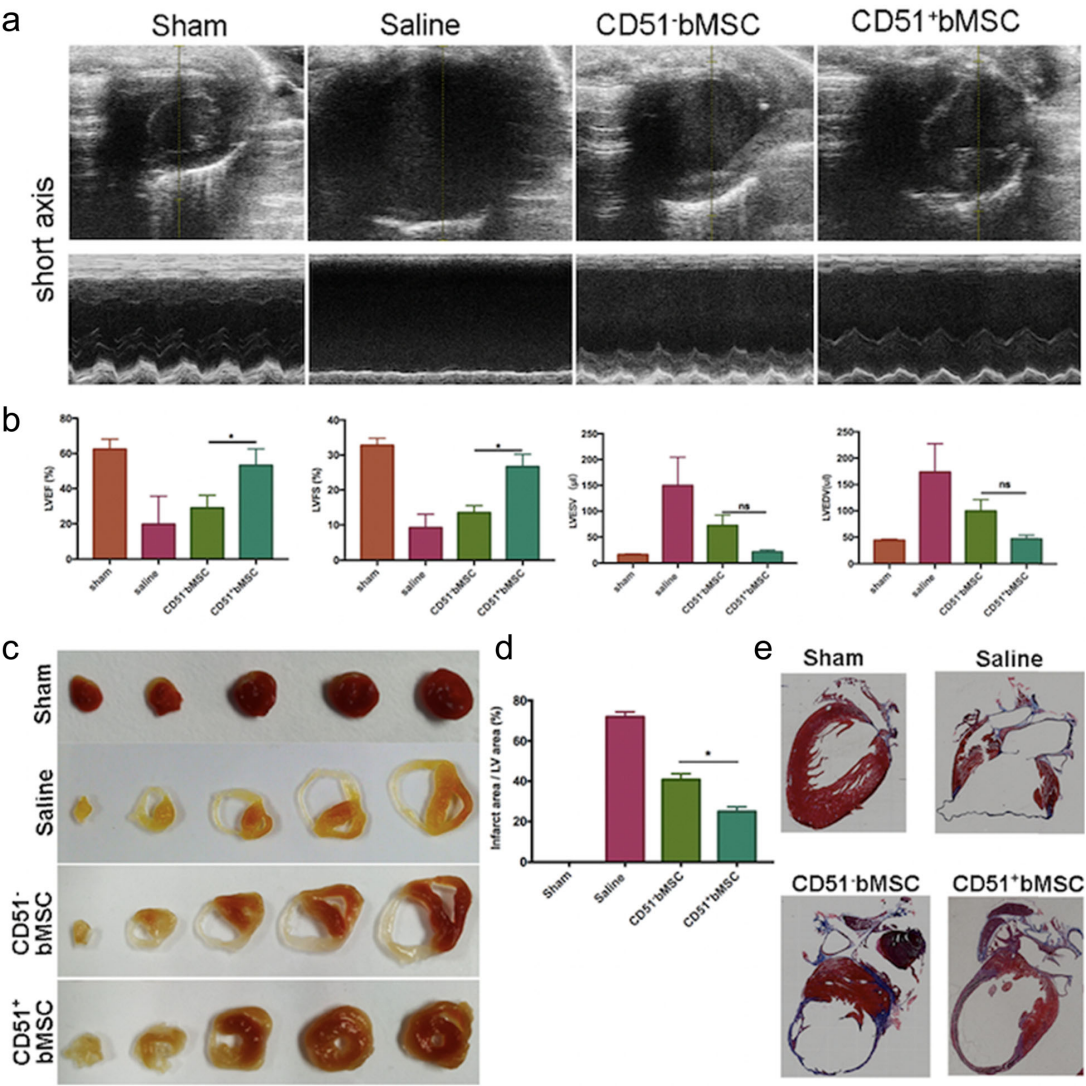
Comparison of cell-based therapeutic efficiency mice with MI. **a** Representative M-mode echocardiographic images of sham-operated, saline-treated, CD51^−^bMSC-treated and CD51^+^bMSC-treated mice. **b** Heart function analysis; quantification of LVEF, LVFS, LVESV and LVEDV (*n* = 3 or 4). **c** Triphenyl tetrazolium chloride (TTC) staining of the heart sections (short axis) at 21 days after treatment. **d** The proportion of the infarct myocardium area was calculated as the percentage of the left ventricular area (the total area of the five heart sections were used for quantification, *n* = 3). **e** Masson’s trichrome staining of the heart sections (long axis) at 21 days after treatment. Data in all panels are presented as the mean ± SD, ns *P* > 0.05, *P < 0.05. LVEF, left ventricular (LV) ejection fraction; LVFS, LV fractional shortening; LVESV, LV end-systolic volume; LVEDV, LV end-diastolic volume

To determine the therapeutic effect on ischaemic injury, we injected cells into the mice immediately after AMI through the tail vein. Two mice died during surgery, and no mice died after surgery. Heart function was monitored by M-mode cardiac ultrasonography at 21 days after treatment. Images exhibited short-axis views of the LV, and M-mode images displayed the cardiac function that was recorded during the end-systolic phases and end-diastole phases (Fig. 3a). As expected, the structure and function of the hearts were normal in the sham-operated group but abnormal in the other three groups, especially in the saline-treated control groups. In particular, we compared heart function between the CD51^−^bMSC and CD51^+^bMSC groups, as illustrated by the left ventricular ejection fraction (LVEF), fractional shortening (LVFS), end-systolic volume (LVESV) and end-diastolic volume (LVEDV) (Fig. 3b). Compared with the CD51^−^bMSC groups, there was a significant improvement in LVEF in the CD51+bMSC groups (28.9% ± 4.1% vs 53.2% ± 5.4%, *P* < 0.05). Additionally, the recovery of LVFS differed significantly between the two groups (13.5% ± 2.0% vs 26.6% ± 3.5%, *P* < 0.05). A short-axis assessment of LVESV at the end of systole revealed less dilation in the CD51^+^bMSC groups than in the CD51^−^bMSC groups, but the data did not show a significant difference (72.4 μl ± 20.3 μl vs 21.7 μl ± 3.8 μl, *P* > 0.05). Consistently, the ventricular diastole volume in the CD51^+^bMSC group showed a minor expansion when compared to that in the CD51^−^bMSC group, but the difference was not significant (99.5 μl ± 21.7 μl vs 46.7 μl ± 7.2 μl, *P* > 0.05). These findings highlighted the idea that cell transplantation promoted cardiac function recovery in the context of MI and that the effect of CD51^+^bMSCs was better than that of CD51^−^bMSCs.

### Cell-based therapy reduced ventricular remodelling after AMI

To determine whether cell delivery was effective in protecting viable myocardium, we adopted TTC staining of heart short-axis sections 21 days after MI. As shown in Fig. 3c, the infarct areas were pale, and the red sites were the viable heart muscles. Quantitative analysis of the proportion of the infarct myocardium area proportion is represented as a percentage of the left ventricular area (total area of the five heart sections was used for quantification). The data demonstrated that the ratio of the infarcted myocardium in the CD51^+^bMSC group was significantly attenuated compared with that of the CD51^−^bMSCs group (24.95% ± 2.33% vs 40.79% ± 2.91%, *P* < 0.05) (Fig. 3d). We also performed Masson trichrome staining of the long-axis heart sections at 21 days post-treatment to confirm the attenuation of fibrosis. Images indicated that all infarcted hearts exhibited scar tissue formation, and the thickness of the infarcted LV in the CD51^+^bMSC groups was better than that in the CD51^−^bMSC groups (Fig. 3e). Notably, CD51^+^bMSC therapy showed more benefits than CD51^+^bMSC therapy in reversing heart remodelling.

### Lentiviral transduction of cells and tracing in vivo

**Fig. 4:**
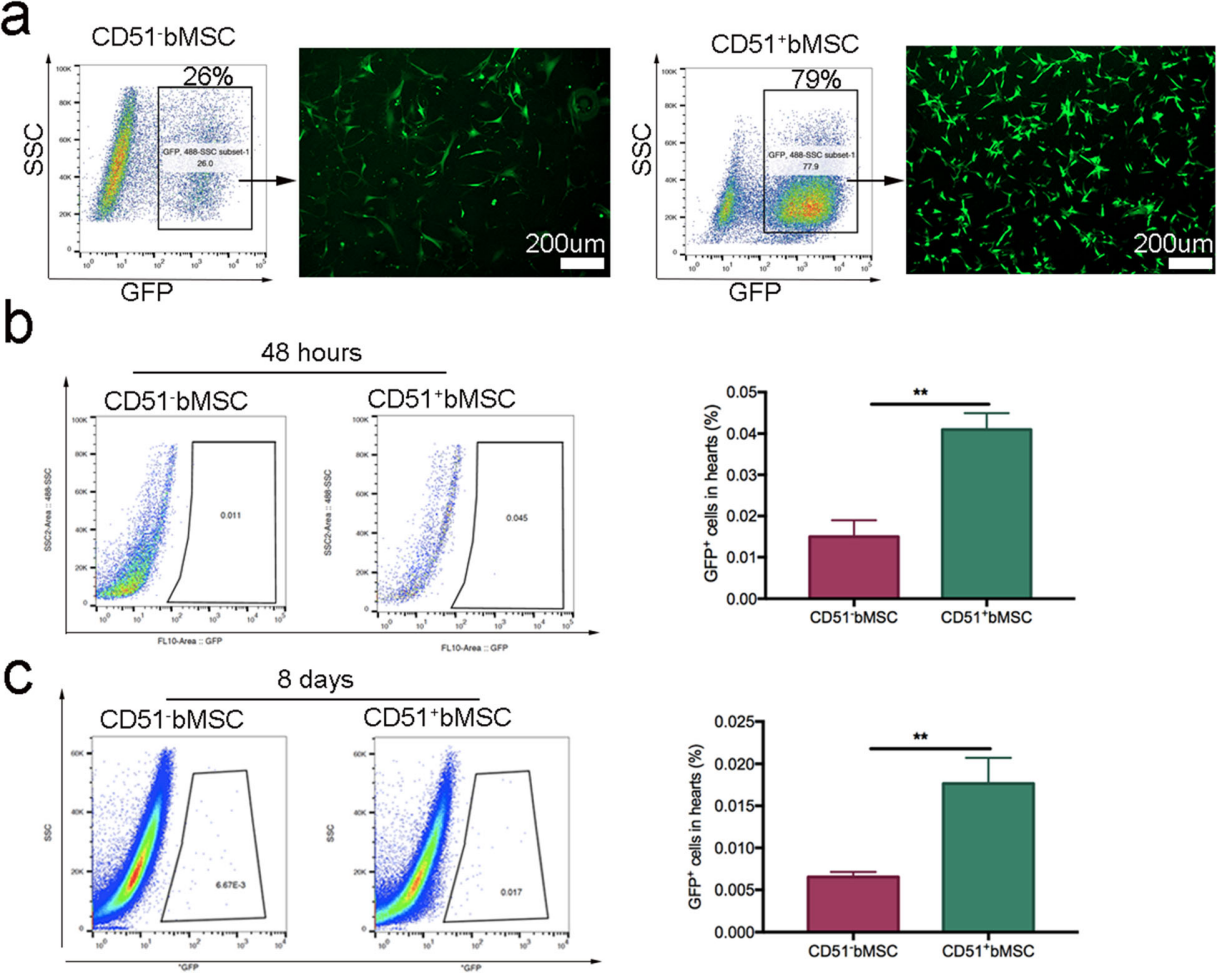
Cell labelling with green fluorescent protein (GFP) and tracing in vivo*.* **a** Lentivirus-infected cells purified by GFP expression using flow cytometry and cultured in the medium. **b**, **c** Measurement of transplanted GFP-positive cells in the whole hearts at 48 h and 8 days after engraftment (*n* = 3). Data in all panels are presented as the mean ± SD, **P < 0.01. Scale bars are marked in the figure

Lentiviral transduction is commonly used to mark cells with colours that can be visualized under a fluorescence microscope or by flow cytometry. Therefore, we infected cells with a recombinant lentivirus that carried a GFP reporter. After subculture two times following the lentiviral transduction, the cells were collected for flow cytometry analysis and purification. As shown in Fig. 4a, the proportion of CD51^−^bMSCs expressing GFP was approximately 26%, and the ratio of GFP-positive CD51^+^bMSCs was approximately 79%. The fluorescence luminance of purified GFP-positive CD51^+^bMSCs was brighter than that of CD51^−^bMSCs. Then, the purified cells were injected into the mice with MI through the tail vein, and the ratio of GFP-positive cells in the heart was analysed by flow cytometry 48 h and 8 days after transplantation. At 48 h, the frequency of migrated CD51^+^bMSCs in the hearts was significantly higher than that of the CD51^−^bMSCs (0.041% ± 0.002% vs 0.015% ± 0.002%, *P* < 0.01) (Fig. 4b). Not surprisingly, the migrated cells in the hearts declined at 8 days after transplantation, and the proportion of CD51^+^bMSCs was still higher than that of CD51^−^bMSCs (0.018% ± 0.0017% vs 0.006% ± 0.0003%, *P* < 0.01) (Fig. 4c). In short, more CD51^+^bMSCs than CD51^−^bMSCs migrated to the injured hearts at both 48 h and 8 days.

### Migration of transplanted cells in vitro

**Fig. 5:**
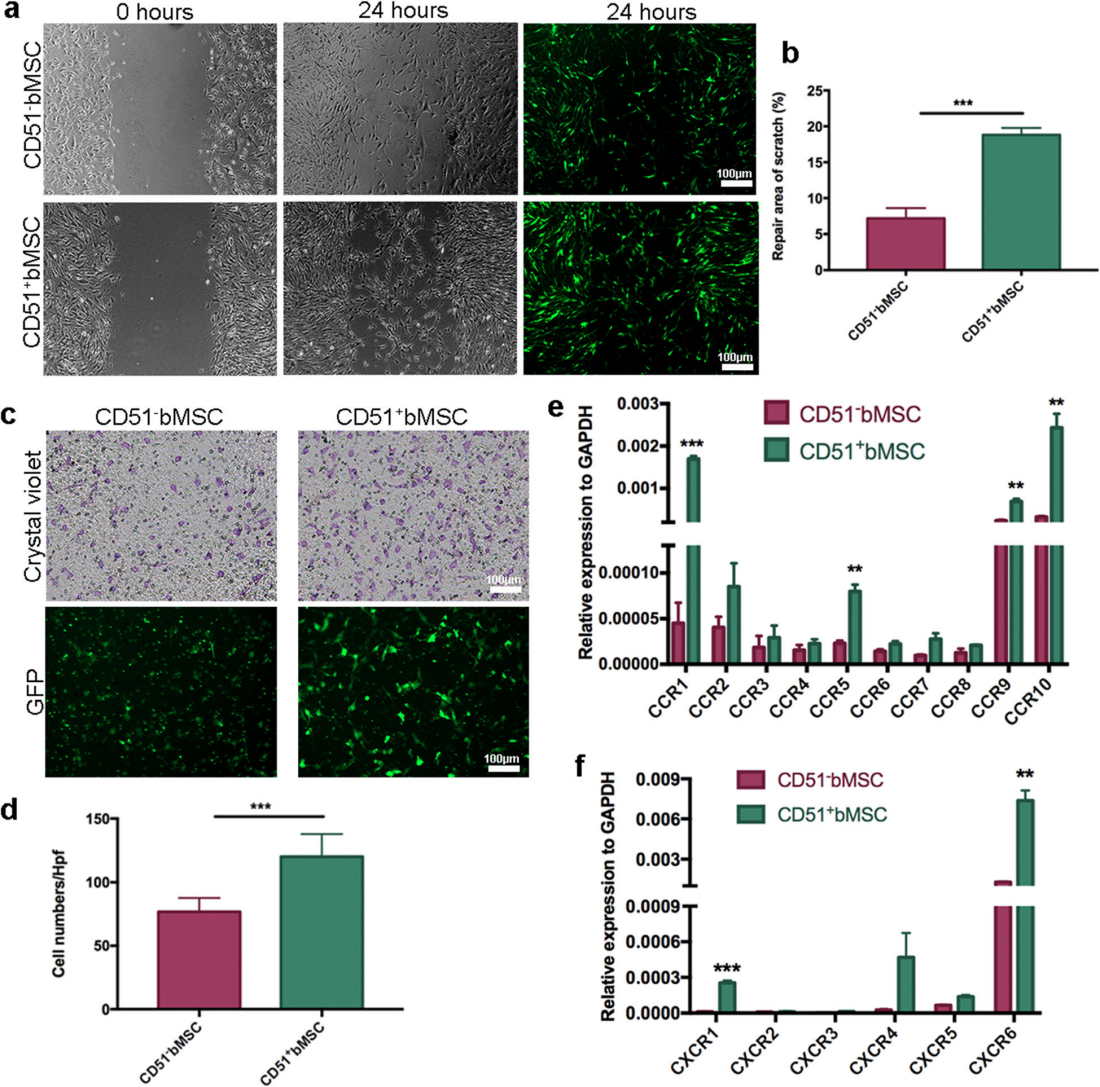
Migratory capacity of CD51^−^bMSCs and CD51^+^bMSCs in vitro. **a**, **b** Images of scratch-wound cell migration assays at 0 h and 24 h; the relative repair area was determined by the total area of migrated cells in the wounds (*n* = 6). **c**, **d** Transwell assay; the migratory cells were fixed at 8 h after plating and stained with crystal violet. Bright-field colour images and fluorescence images were randomly taken, and the cell numbers were calculated (*n* = 8). **e**, **f** Relative mRNA expression of the chemokine receptors; the gene expression levels were normalized to GAPDH (*n* = 3). Data in all panels are presented as the mean ± SD, **P < 0.01, **P < 0.001. Scale bars are marked in the figure

To determine whether the migratory capacity of CD51^+^bMSCs was better than that of CD51^−^bMSCs, we performed scratch assays and transwell assays. Images of the scratch assay demonstrated that many CD51^+^bMSCs migrated to the scratch wound, whereas fewer CD51^−^bMSCs migrated (Fig. 5a). The migrated cells captured by phase-contrast microscopy were consistent with the fluorescence microscopy results; thus, we calculated the total area of migrated GFP-positive cells in the wound to determine the relative repair area. The repair area percentage of CD51^+^bMSCs was significantly higher than that of CD51^−^bMSCs (18.81% ± 0.39% vs 7.19% ± 0.57%, *P* < 0.001) (Fig. 5b). Accordingly, numerous CD51^+^bMSCs migrated outside of the filters that were visualized by either crystal violet staining or direct GFP fluorescence (Fig. 5c). The numbers of migrated CD51^+^bMSCs were significantly elevated compared with those of CD51^−^bMSCs (120 ± 6 vs 76 ± 3, *P* < 0.001) (Fig. 5d). In addition, we analysed the gene expression of chemokine receptors, including CCR families and CXCR families, by PCR. CCR1 was significantly increased in CD51^+^bMSCs compared to CD51^−^bMSCs, and CCR2, CCR3, CCR4, CCR5, CCR6, CCR7, CCR8, CCR9 and CCR10 were moderately elevated (Fig. 5e). With respect to CXCR, the expression of CXCR1, CXCR2, CXCR3, CXCR4, CXCR5 and CXCR6 also remained higher in CD51^+^bMSCs than in CD51^−^bMSCs (Fig. 5f). In summary, the migratory capacity of CD51^+^bMSCs was greater than that of CD51^−^bMSCs, and the gene expression of the corresponding chemokine receptors was also higher in CD51^+^bMSCs.

### Identification of CD51^+^bMSCs in the hearts

**Fig. 6:**
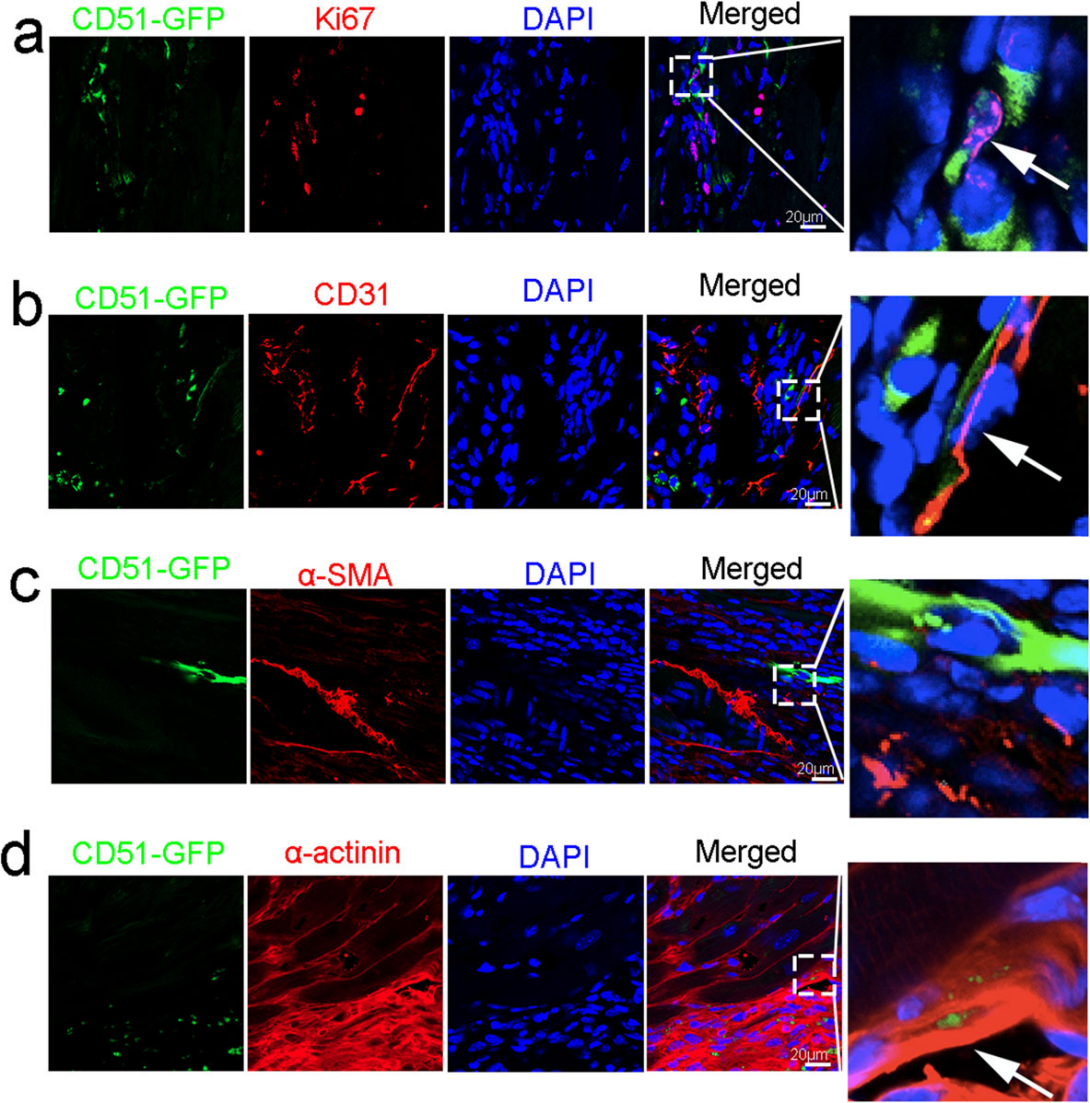
Characteristics of CD51^+^bMSCs in vivo. The hearts were collected for immunofluorescence staining 10 days after transplantation. **a**–**d** The heart sections stained with the listed antibodies Ki67, CD31, α-SMA and α-actinin (red). 4′,6′-Diamidino-2-phenylindole (DAPI, blue) was used to stain the nuclei. GFP-positive CD51^+^bMSCs are green. Scale bars are marked in the figure

To characterize the implanted CD51^+^bMSCs in the hearts, we produced the heart sections 10 days after transplantation for immunofluorescence staining. First, the heart sections were stained with Ki67 to investigate CD51^+^bMSC proliferation. CD51^+^bMSCs survived in the cardiac tissue, and some of them proliferated, as confirmed by Ki67 staining (Fig. 6a). Second, we stained the hearts with the endothelial cell marker CD31 to examine the endothelialization capacity of CD51^+^bMSCs. Many endothelial cells surrounded the CD51^+^bMSCs, but only a few GFP-positive cells were colocalized with CD31-positive cells (Fig. 6b). As α-smooth muscle actin (α-SMA) is a marker of smooth muscle cells or fibroblastic cells, we performed α-SMA staining on cardiac sections to evaluate the differentiation of CD51^+^bMSCs to these cell clusters. As shown in Fig. 6c, there was a little colocalization between GFP and α-SMA in both blood vessels and interstitial tissues. To study the relationship between CD51^+^bMSCs and heart muscles, we stained sarcomeric α-actinin, which is a microfilament protein expressed in the cardiac muscle. The results showed that CD51^+^bMSCs remained near the *z*-disc that stained with α-actinin (Fig. 6d). Unexpectedly, CD51^+^ bMSCs could partially differentiate into endothelial cells (Vwf) and smooth muscle cells (α-SMA) under both hypoxic and normoxic conditions in vitro, and most cells expressed the myocyte marker sarcomeric α-actinin (Additional file 1: Figure S2). Thus, the injected CD51^+^bMSCs could survive and proliferate in cardiac tissues, but they only differentiated into endothelial cells, smooth muscle cells, fibroblastic cells and cardiomyocytes.

### CD51^+^bMSC therapy attenuated inflammatory responses in the hearts after MI

**Fig. 7:**
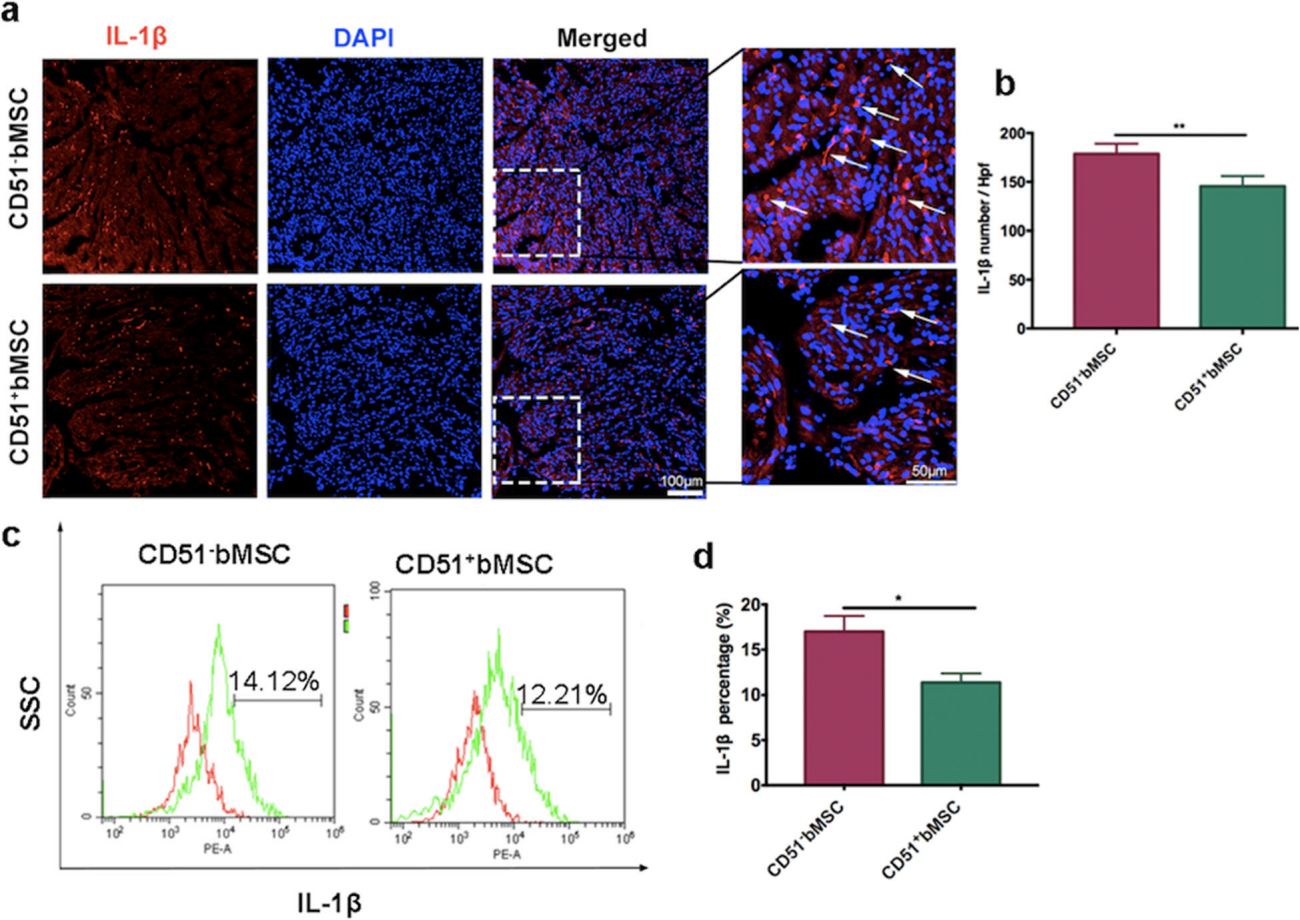
CD51+bMSC therapy attenuated inflammatory responses in the hearts after MI. **a** Immunofluorescence staining of IL-1β (red) in the border areas of the heart sections from the CD51^−^bMSC- and CD51^+^bMSC-treated mice at day 3 after MI. DAPI was used to stain the nuclei. Scale bars are marked in the figure. **b** Quantification of IL-1β-positive cells is presented as the percentage per high power field (Hpf) (*n* = 5). **c** Representative images of IL-1β expression was determined by flow cytometry in the hearts from cell-treated mice on day 3 after MI. Red histogram is the negative control. The left side of the green histogram (in the negative control part) represents the negative expression of IL-1β marker; the right side represents the positive expression of IL-1β marker. **d** Measurement of IL-1β-positive cells is presented as the percentage of the total cells in the injured ventricle on day 3 after MI (*n* = 3). Data in all panels are presented as the mean ± SD, *P < 0.05, **P < 0.01

Chemokine receptors play an important role in inflammatory modulation. To verify the treatment mechanism of CD51^+^ bMSCs in acute MI, we collected the infarcted hearts at 3 days post-cellular transplantation to test the expression of IL-1β by immunofluorescence staining and flow cytometry analysis. The expression of IL-1β in the injured hearts was significantly decreased in the CD51^+^bMSC-treated groups compared with the CD51^−^bMSC-treated groups (Fig. 7a, b). Similarly, flow cytometry showed that the percentage of IL-1β-positive cells among the total injured ventricular cells was reduced in the CD51^+^bMSC-treated groups compared with the CD51^−^bMSC-treated groups (Fig. 7c, d). Additionally, the gene expression of IL6 in CD51^−^bMSC-treated hearts were also significantly higher than that in CD51^+^bMSC-treated hearts (Additional file 1: Figure S3). In summary, CD51^+^bMSC therapy attenuated the inflammatory responses in the hearts after MI.

## Discussion

CD51 is a critical marker for distinguishing a promising subpopulation of bMSCs based on the following findings. (1) The isolated CD51^−^bMSCs and CD51^+^bMSCs showed a proliferative capacity and multi-differentiation potential. (2) The therapeutic efficiency of CD51^+^bMSCs was better than that of CD51^−^bMSCs in mice with MI. (3) Compared with CD51^−^bMSCs, CD51^+^bMSCs preferentially migrated to and were retained in the infarcted hearts of mice both at 48 h and 8 days after intravenous injection. (4) The migration capacity of CD51^+^bMSCs also exceeded that of CD51^−^bMSCs in vitro, yet the expression of chemokine receptors or ligands was higher in the former population. (5) The CD51^+^bMSCs retained in the infarcted hearts possessed proliferative potential but could only differentiate into only parenchymal cells, such as endothelial cells, smooth muscle cells, fibroblasts and cardiomyocytes. Therefore, the present study demonstrated that the migration potential of CD51^+^bMSCs played an important role in the repair of MI in the heart.

The percentage of CD51-positive MSCs in the bone marrow of 14-day-old mice was approximately 14.4% (data not shown) but dramatically declined to 1.4% after excluding haematopoietic cells, endothelial cells and erythroid cells (defined as CD51^+^bMSCs). Therefore, extensive expansion of CD51^+^bMSCs in vitro was essential for the further application of cell transplantation, which required sufficient numbers of cells. A high FBS concentration (10% FBS in the standard culture medium) resulted in cellular senescence when the cells underwent multiple passages for a long period. Hence, we reduced the proportion of FBS to 2% in the supporting culture medium and added supplements, including B27, EGF and bFGF, to maintain the potency of the stem cells. The number of CD51^+^bMSCs quickly increased within 24 h, which indicated their high capacity for expansion. The proliferative potential of CD51^+^bMSCs was maintained under hypoxic-ischaemic conditions in vitro, but these conditions would accelerate cellular apoptosis.

We ascertained that CD51^+^bMSCs displayed an enhanced migratory capacity in vitro.

Chemokine receptors are a subfamily of G-protein-coupled receptors that play an important role in chemotactic migration and inflammatory modulation. At present, researchers have shown that chemokine receptors are critical for the migration and recruitment of stem cells [24–27]. Chen et al. demonstrated that tumour necrosis factor (TNF)-α primed MSCs could stimulate the proliferation and progression of colon cancer cells via the CCl5/CCR1/β-catenin/Slug pathway [28]. Additionaly, after revert to a primitive stem cell population, bone marrow stromal cells displayed an enhanced homing capacity to home tumors, which was associated with the activation of the CCL5/CCR1/ERK signaling pathway [29]. Piryani et al. found that the survival potential of murine haematopoietic cells was improved after radiation, which was due to the increase in CCR5 expression [30]. CCR9 could enhance the proliferative and invasive ability of cancer stem cells and helped to recruit bone marrow-derived progenitors into the thymus [31, 32]. Similarly, the downregulation of CCR10 tumour cells contributed to the inhibition of cancer stem cell growth and metastases in both melanoma and squamous cell carcinoma [33]. Accordingly, the expression of chemokine receptors, especially CCR1, CCR5, CCR9, CCR10, CXCR1 and CXCR6, was elevated in CD51^+^bMSCs in our study. As suggested by Du, CXCR1 expressed on osteosarcoma cells maintained the viability and metastasis of cancer stem cells via the IL-8/CXCR1/Akt pathway [34]. Additionally, Blaser et al. showed that CXCR1 positively participated in the regulation of haematopoietic stem and progenitor cell colonization and improved the therapeutic efficiency of cell engraftment [35]. As reported by Jung et al. CXCR6 was regarded as one of the mechanisms for epithelial-to-mesenchymal transition and metastasis in prostate tumour cells [36]. Thus, we concluded that the strong migratory capacity of CD51^+^bMSCs was associated with the high expression of chemokine receptors.

Considering that chemoattractant molecules and adhesion molecules were elevated in the ischaemic myocardium, CD51^+^bMSCs preferentially migrated to the heart due to the high expression of many chemokine receptors. Intravenous administration is a convenient route for repeated delivery of MSCs, but the low migratory rate limited its utility. Kraitchman et al. demonstrated that radiotracer-labelled allogeneic mesenchymal stem cells were already localized to the target infarcted and non-target normal myocardium at 24 to 48 h after intravenous injection and persisted for 7 days [37]. Accordingly, our data clearly showed that the GFP-labelled CD51^+^bMSCs were distinctly observed at 48 h after infusion and were still detected at 8 days after infusion. Therefore, transplantation of CD51^+^bMSCs by intravenous injection is appropriate, and the cells showed moderate localization and survival in myocardial tissue. Here, 10-month-old mice were selected to establish the MI model, which was coincident with age-associated clinical morbidity. To maintain the uniformity of the injured individuals, mice of similar weights were selected for the experiments, the ligation site was strictly fixed at the same point of the left anterior descending branch and the experiment was designed with random allocation of the animals after MI. In addition, some reports demonstrated that combination treatment with statins, biomaterials or biological effectors may improve the retention of cells in the hearts.

In mice, we confirmed that CD51^+^bMSC delivery attenuated the ischaemic zone in the infarcted hearts and reduced myocardial fibrosis, thereby improving cardiac function to a moderate extent. The multiple characteristics of CD51^+^bMSCs could provide auxiliary results to MI in vivo*.* Our data demonstrated that the proliferative fate of transplanted CD51^+^bMSCs could be clearly detected in the hearts at 10 days after delivery. Nagaya et al. indicated that some engrafted bMSCs in the hearts were positively stained for markers of the myocardium, endothelial cells and smooth muscle cells in dilated cardiomyopathy [38]. Igura et al. confirmed that bone marrow-derived small juvenile stem cells could differentiate into cardiomyocytes in vivo [39]. However, we found that CD51^+^bMSCs hardly differentiated into endothelial cells, smooth muscle cells or cardiomyocytes in the ischaemic hearts. In vitro, CD51^+^ bMSCs could partially differentiate into endothelial cells, smooth muscle cells and myocytes, but not fibroblasts, under both hypoxic and normoxic conditions. This difference was not surprising. Finally, we found that CD51^+^bMSCs were capable of modulating the inflammatory response in the injured heart, and further studies are ongoing to explore the exact molecular mechanisms by which CD51^+^bMSCs repair the injured hearts.

## Conclusion

The CD51-distinguished subpopulation of bMSCs could be obtained from immature mice and possessed the characteristics of MSCs. The migratory capacity of CD51^+^bMSCs was stronger than that of CD51^−^bMSCs both in vivo and in vitro, which was probably related to the abundant expression of chemokine receptors. The CD51^+^bMSC treatment had more benefits for cardiac healing in mice with MI than did CD51^−^bMSCs after intravenous transplantation, and the effects of the retained CD51^+^bMSCs retained in the hearts might be associated with the anti-inflammatory capacity. Our preliminary results provide further evidence for the utility of CD51^+^bMSCs as a cell-based therapeutic strategy to attenuate the harmful outcomes of MI.

## Supplementary information

**Additional file 1.** Supplementary methods and figures. (DOCX 1275 kb)

## Data Availability

All the data generated or analysed during this study are included in this published article.

## Abbreviations

bMSCs: Bone marrow-derived mesenchymal stromal/stem cells
EdU: 5-Ethynyl-2′-deoxyuridine
qPCR: Quantitative polymerase chain reaction
MI: Myocardial infarction
GFP: Green fluorescent protein
TTC: Triphenyl tetrazolium chloride
LVEF: Left ventricular ejection fraction
LVFS: Left ventricular fractional shortening
cDNA: Complementary DNA
LAD: Left anterior descending coronary artery
LV: Left ventricle
LVESV: Left ventricular end-systolic volume
LVEDV: Left ventricular end-diastolic volume
MOI: Multiplicity of infection
PFA: Paraformaldehyde
FBS: Foetal bovine serum
bFGF: Basic fibroblast growth factor
EGF: Epidermal growth factor
