# Supplementary material for: CD51 distinguishes a subpopulation of bone marrow mesenchymal stem cells with distinct migratory potential: a novel cell-based strategy to treat acute myocardial infarction in mice
Source: Stem Cell Res Ther. 2019 Nov 20;10:331. doi: 10.1186/s13287-019-1439-y (PMC6865070; doi:10.1186/s13287-019-1439-y)
Supplement: Supplementary file 1 — Additional file 1. Supplementary methods and figures. (DOCX 1275 kb) [file 13287_2019_1439_MOESM1_ESM.docx]

Additional file 1

Supplement methods

**Tunel assay**

Apoptosis analysis was performed in CD51^+^bMSCs which was u hypoxia (1% O_2_) or normoxia (5% O_2_) conditions for 24 hours in the presence of the 1% concentrations of FBS. Total terminal deoxynucleotide Tunel-positive cells were assessed in CD51^+^bMSCs.

**Differentiation of CD51^+^bMSCs *in vitro***

To demonstrate the differential capacity of CD51^+^bMSCs under normal and hypoxic-ischemic condition, we cultured cells with conditioned media which induced differentiation into endothelial cells, smooth muscle cells or myocytes, respectively. Hypoxia condition were treated with 1% O_2_ for the first 48 hours, then changed to the normal oxygen concentration (5% O_2_). Cells were cultured in Endothelial growth medium-2 (Lonza, American) for 14 days to induced endothelial cells and tested by Vwf staining. Smooth muscle cells were induced in myogenic medium consisting of fresh DMEM medium with 10% FBS and myogenic growth factors (PDFF-BB, hepatocyte growth factor, and transforming growth factor-b)[1]. After cultured for 14 days, cells were verified by αSMA staining. Myocytes was induced for 7 days in IMDM medium supplemented with 10% FBS and 3μmol/l of 5-azacytidine (Sigma, USA)[2]. Then, they were confirmed by α-actinin staining.


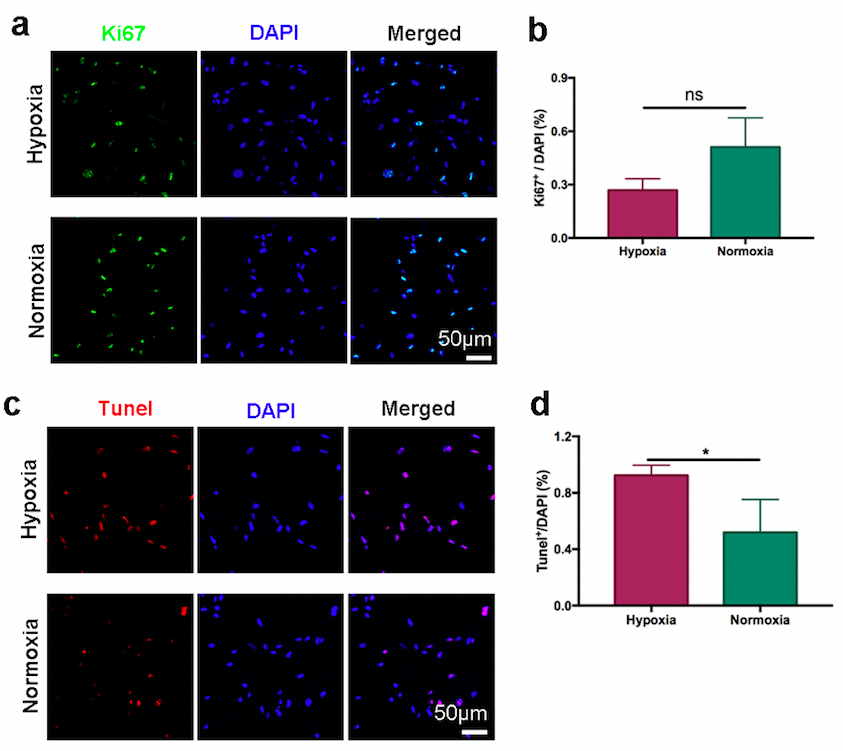


**Figure S1.** **Characteristic of CD51^+^bMSCs under hypoxia or normoxia conditions *in vitro*.** CD51^+^bMSCs were incubated under hypoxia (1% O_2_) or normoxia (5% O_2_) conditions for 24 hours in the presence of the 1% concentrations of FBS. a-b. Representative images of CD51^+^bMSCs labeled with Ki67 using fluorescent immunohistochemistry. Compared with normoxia condition, percentage of cells expressing Ki67 (% total) were decreased under hypoxia condition but there was no significance difference (n=3). c-d. Representative images of CD51^+^bMSCs labeled with Tunel using fluorescent immunohistochemistry. Compared with normoxia condition, percentage of cells stained by Tunel (% total) were significantly increased under hypoxia condition (n=3). DAPI stain were used to indicated the number of total cells. Results expressed as mean ± SD, ns *P*>0.05, * *P*<0.05. The scale bar was marked in the figure.


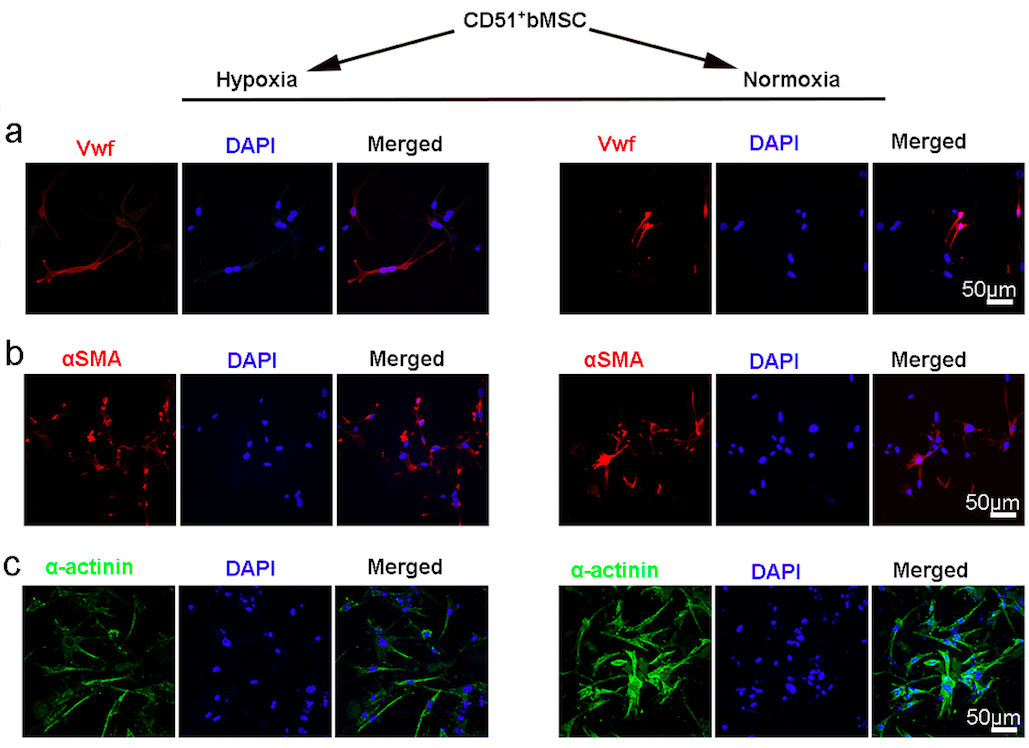


**Figure S2. Differential characteristic of CD51^+^bMSCs under hypoxia or normoxia conditions *in vitro****.* CD51^+^bMSCs were incubated under hypoxia or normoxia conditions in the presence of the indicated medium to induce differentiated into endothelial cells, smooth muscle cells, and myocytes respectively. Hypoxia condition were treated with 1% O_2_ for the first 48 hours, then changed to the normal oxygen concentration (5% O_2_). a-c. Fluorescent immunohistochemistry stain of Vwf, αSMA, α-actinin to indicate endothelial cells, smooth muscle cells, and myocytes, respectively. The scale bar was marked in the figure.


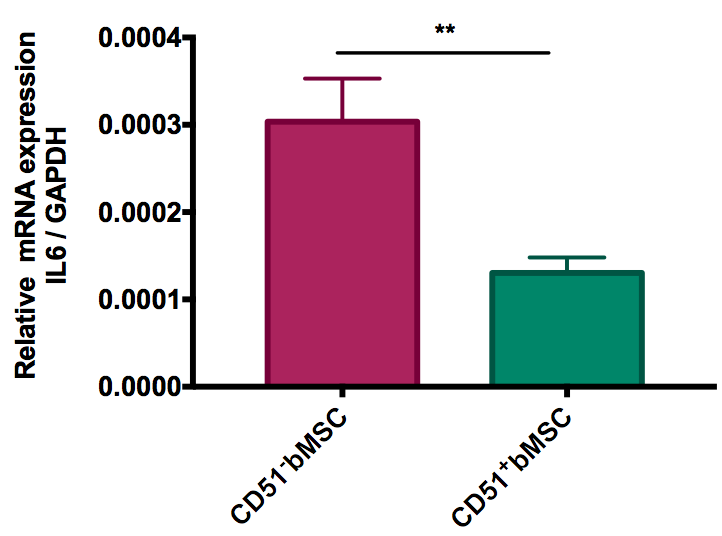


**Figure S3. mRNA expression of IL6 in hearts after CD51^-^bMSCs and CD51^+^bMSCs treatment.** qPCR quantification of IL6 expression in the injured hearts after CD51^-^bMSC- and CD51^+^bMSC- therapy at day 3 after MI. Data in all panels are presented as the mean ± SD (n=6), ***P* < 0.01.

1. Tian, H., et al., *Differentiation of human bone marrow mesenchymal stem cells into bladder cells: potential for urological tissue engineering.* Tissue Eng Part A, 2010. **16**(5): p. 1769-79.

2. Makino, S., et al., *Cardiomyocytes can be generated from marrow stromal cells in vitro.* J Clin Invest, 1999. **103**(5): p. 697-705.
